# Multiscale knowledge distillation with attention based fusion for robust human activity recognition

**DOI:** 10.1038/s41598-024-63195-5

**Published:** 2024-05-30

**Authors:** Zhaohui Yuan, Zhengzhe Yang, Hao Ning, Xiangyang Tang

**Affiliations:** Department of Software Engineering,School of Software, East China Jiaotong University, No. 808 Shuanggang East Street, Nanchang, 330013 Jiangxi China

**Keywords:** Human activity recognition, Knowledge distillation, Multi-modalities, Self-attention, Transfer learning, Information theory and computation, Computational science, Computer science

## Abstract

Knowledge distillation is an effective approach for training robust multi-modal machine learning models when synchronous multimodal data are unavailable. However, traditional knowledge distillation techniques have limitations in comprehensively transferring knowledge across modalities and models. This paper proposes a multiscale knowledge distillation framework to address these limitations. Specifically, we introduce a multiscale semantic graph mapping (SGM) loss function to enable more comprehensive knowledge transfer between teacher and student networks at multiple feature scales. We also design a fusion and tuning (FT) module to fully utilize correlations within and between different data types of the same modality when training teacher networks. Furthermore, we adopt transformer-based backbones to improve feature learning compared to traditional convolutional neural networks. We apply the proposed techniques to multimodal human activity recognition and compared with the baseline method, it improved by 2.31% and 0.29% on the MMAct and UTD-MHAD datasets. Ablation studies validate the necessity of each component.

## Introduction

To overcome the limitations of traditional machine learning with single-modal data, adopting multimodal data machine learning has become an important method for enhancing the performance of deep learning models. However, traditional multimodal data learning models require the synchronous input of various modal data for training and prediction, which is not always possible in many real-world application scenarios. For example, in conventional monitoring applications, it is often difficult to synchronize motion sensor data with the motion images of a monitored object in real time, leading to a significant decline in the performance of multimodal models, and sometimes the accuracy is even lower than that of models trained with single-modal data.

Correspondingly, to improve the robustness of multimodal machine learning algorithms mentioned above, knowledge distillation technology^1^ offers an effective solution. Generally, knowledge distillation techniques train different models with data from different modalities, defining these models as student and teacher models, respectively. During training, the student acquires knowledge from the teacher, thereby fully utilizing the knowledge from multiple modalities. The advantage of this mechanism is that during the prediction or recognition phase, the student model can utilize both its own knowledge and that learned from the teacher model to achieve predictions that are close to or even better than those with multimodal data, even in the presence of only unimodal data. Although knowledge distillation technology has been successfully applied in many fields, traditional knowledge distillation frameworks still face the following problems:

- Traditional knowledge distillation structures usually only transfer knowledge at the final feature output layer, ignoring the extraction of knowledge at intermediate feature layers of different scales. This may lead to the student network not fully learning complementary knowledge from the teacher network, which is limited in its receptive field of data features, and thus reducing the efficiency of multimodal learning.
- In traditional knowledge distillation, the data of teacher modality modality generally considers only a single data type, neglecting the fusion representation of multiple different types of data. For example, in multimodal human activity recognition (HAR), when training a teacher network with wearable sensor sampling data, the data from wearable sensors will include various physically different data such as acceleration, gyroscope, orientation, and even magnetometer readings, requiring a specific fusion mechanism to fully utilize the knowledge of correlations within these data.
- In traditional knowledge distillation, both teacher and student networks typically use convolutional neural networks (CNNs) as their backbone, but convolutional neural networks lack globality in early feature extraction. This limits the model’s ability to extract good features, thereby restricting the system’s ultimate performance.

To address these obstacles, we propose a new multiscale knowledge distillation architecture that overcomes the shortcomings of traditional knowledge distillation techniques, and selects human activity recognition (HAR) as the target problem. Specifically, against the backdrop of teacher networks transferring knowledge to student networks at only a single scale layer, we propose multiscale semantic graph mapping (SGM) knowledge distillation loss at different network nodes. This module uses semantic embedding for joint representation of different types of data and combines the internal graph structure of final feature generation to generate semantic change rates at corresponding scales, ultimately generating focus areas on intermediate feature maps of different modalities. We calculate the knowledge distillation loss based on the multiscale differences in the focus areas of intermediate feature maps between teacher and student networks to complete the transfer of knowledge from the teacher to student networks at different intermediate layers. This facilitates more comprehensive learning of heterogeneous knowledge by student networks from intermediate layers of teacher networks.

Furthermore, for the joint representation of different types of data within a modality, we designed an Fusion and Tuning (FT) module. This module inputs different types of data into multiple teacher networks and fuses multiple data features of teachers within the teacher network to calibrate the original features among various teachers’ data, ensuring similarity within and correlation between different types of data. This allows for the full utilization of complementary information from different types of data, and extracting knowledge is more beneficial for learning of student networks.

Additionally, to address the issue of incomplete feature extraction by early convolutional neural networks, we introduce a self-attention mechanism and adopt the Swin Transformer^2^ as the backbone network for teacher networks while introducing Video Swin Transformer^3^ as the backbone for student networks. The self-attention mechanism enables global analysis of relationships between data at an early stage of feature extraction, aiding in better extraction of useful information and laying a solid foundation for subsequent knowledge transfer. Finally, we constructed our model based on features from the MMAct^4^ and UTD-MHAD^5^ HAR datasets and designed extensive experiments to validate the effectiveness of our proposed structure and methods. Our results demonstrate performance improvements over other traditional knowledge distillation models and, employ corresponding ablation studies to analyze the necessity of our proposed structures. The contributions of this paper are as follows:

- We designed a new multiscale knowledge distillation loss function, semantic graph mapping (SGM) loss, based on feature outputs at different scale stages to calculate multiscale knowledge distillation loss between teacher and student networks, achieving more comprehensive knowledge transfer from teacher to student networks.
- To fully utilize knowledge from different sensor sampling data within a modality, we designed a fusion and tuning (FT) module for fusing features of different types of data and correcting features across modules to maintain similarity within single modalities and complementarity between modalities.
- We selected the HAR problem, designed extensive experiments on different datasets, and compared our model with current research findings. Our proposed model outperforms the baseline algorithm on both the MMAct and UTD-MHAD datasets. These results validate the effectiveness of the proposed architecture.

## Related work

### Unimodal activity recognition

Human activity recognition is an active research field^6^, in which traditional methods mainly rely on a single modality and can be broadly categorized into three types: (1) Handcrafted representation-based methods^7–14^. (2) Graph-based learning methods^15–22^. and (3) Deep learning-based methods^23–33^. It is noteworthy that these methods largely rely on visual sensor modalities such as skeletal, infrared imagery, depth maps, or RGB videos. This indicates that image-based methods are generally more intuitive in recognizing human activities. Wang et al.^34^ proposed a method using segment sampling and aggregation modules to model remote temporal structures, implying that when processing video data, it is unnecessary to handle all frames, and only selecting key frames sufficient. Zhou et al.^35^ achieved higher accuracy in video classification by learning and inferring time dependencies across multiple time scales of video frames, emphasizing the crucial role of temporal correlations between video frames in video classification. Li et al.^36^ introduced a spatio-temporal manifold network (STMN), which utilizes data manifold structures to regularize deep action feature learning, investigating the influence of structural properties within the data on feature learning. Luo et al.^37^ proposed a semantic-assisted network for video action recognition, indicating that semantic information can bridge the conceptual gap between raw video data and human behaviors. Qiao et al.^38^ proposed a federated learning method to solve the problem of user customization. Ren et al.^39^ proposed GSKG to make data acquisition more secure. Although the aforementioned methods have achieved significant advancements in segment sampling, considering temporal relations between frames, data stream structures, and semantic assistance, they fail to address issues that are challenging for images to handle, such as cluttered backgrounds, occlusions, and varying camera perspectives. Therefore, alternative approaches are needed to address these challenges.

With the widespread adoption of wearable sensors such as smartphones and smartwatches, action recognition based on wearable sensors has become a key research direction in human activity understanding^40^. Many issues inherent in vision-based approaches can be effectively addressed through wearable sensor data. This is because wearable sensor data primarily focus on capturing detailed variations in motion, unaffected by surrounding visual environmental changes. Zebhi et al.^41^ transformed motion sensor data into images using two-dimensional fast fourier transform (2-D FFT) and wigner-ville transform (WVT) to achieve human activity recognition, enhancing the representation capability of sensor data by converting one-dimensional time-series signals into images. To improve accuracy, Alghamdi et al.^42^ applied a multi-head attention mechanism to realize two-level interaction based on inertial sensors. Shi et al.^43^ first learned features from different sensors and then used Transformer encoders to model dependencies between multisensor data and extract features. Both approaches indicate that sensor data between different modalities contain complementary information that can be leveraged. Wannenburg et al.^44^ utilized ten different classification algorithms to classify human behaviors using accelerometer signals captured by smartphones. Unlike visual sensor-based approaches, the data format of wearable sensor data is typically a one-dimensional time series. Compared to image data, wearable sensor data inherently lack color, texture, and other essential information. This makes it challenging to accurately identify certain specific actions that require visual resolution, such as making a fist and waving versus opening the palm and waving.

Combining the characteristics of the above methods, to enhance the representation capability of wearable sensor data features, we adopted gramian angular field^45^ (GAF) technique to transform one-dimensional time-series signals into RGB images. To fully utilize the feature representation capability of different sensor modalities, we fused and corrected features from different sensors during the intermediate feature extraction process to maintain similarity between different modalities.

### Multimodal activity recognition

While single-modality action recognition methods have achieved decent recognition accuracy, there are still many limitations, necessitating richer data to enhance model robustness. With the development of hardware devices such as deep learning device, cameras, and wearable devices, an increasing number of multimodal action recognition methods have emerged in the field of human motion recognition. Chen et al.^46^ directly input data from different modalities into a Transformer as sequences for action recognition. Zhong et al.^47^ utilized skeletal and depth data to design a motion co-occurrence spatial feature model, integrating information from various joints and proposing a quantification standard to measure the contribution of each joint movement to the recognition results. Bruce et al.^48^ devised a model-level multimodal fusion approach, employing spatiotemporal graph convolutional networks as the skeletal modality to learn how to transfer attention weights from the skeletal modality to the RGB modality network. Shaikh et al.^49^ proposed a meaningful representation extraction method for image and audio and fused it with video representation, demonstrating better performance in activity recognition compared to single-modality audio and video. The first two methods primarily rely on multihead self-attention mechanism-based multimodal recognition methods, enabling the global learning of relationships between different modalities. The latter two methods employ traditional convolutional neural network methods, with Bruce separately learning knowledge from different models for each modality and then combining it, while Shaikh transfers knowledge learned from audio to RGB videos using attention weights.

Unlike the aforementioned multimodal methods, we adopt a knowledge distillation architecture to leverage multimodal data, allowing the teacher network to transfer knowledge to the student network. Ultimately, only inputting data from one modality can yield results approximating those from multimodal data.

### Knowledge distillation in HAR

**Figure 1:**
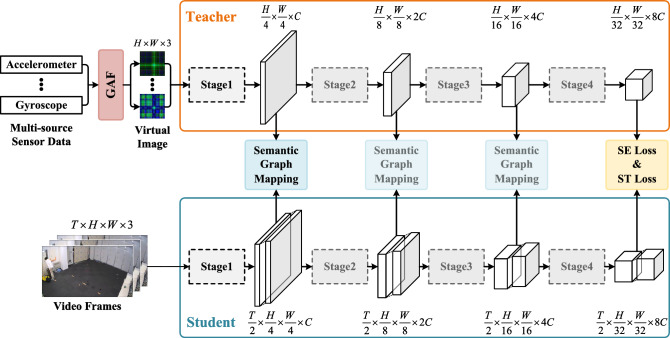
The workflow of the proposed framework. Sensor data is transformed into virtual images through GAF and serves as input to the teacher network. The student network takes video frame sequences as input. Both the teacher and student networks consist of four stages, with knowledge distillation conducted between the feature maps outputted at each stage.

Knowledge distillation is a general technique used to supervise the training of student networks by capturing and transferring useful knowledge from well-trained teacher networks. Hinton et al.^1^ employed softened labels with temperature to transfer knowledge to smaller student networks. Ni et al.^46^ proposed a novel progressive Skeleton sensor knowledge distillation (PSKD) model, which combines acceleration with the corresponding skeleton positions for better representation. Xu et al.^48^ introduced a novel contrastive distillation with regularization knowledge (ConDRK) framework, which can significantly eliminate biases introduced by teachers. Deng et al.^47^ presented a lightweight human activity recognition (LHAR) knowledge distillation framework, allowing the model to balance performance and complexity through multiple depth-wise separable convolutions. Park et al.^50^ introduced distance and angle distillation losses to achieve relational knowledge transfer. Tung et al.^51^ constructed a knowledge distillation loss, the constraint of which is to generate similar (dissimilar) activations in the teacher network corresponding to inputs that should generate similar (dissimilar) activations in the student network. Crasto et al.^52^ introduced feature-based losses compared to flow, specifically a linear combination of feature-based loss and standard cross-entropy loss, to mimic motion flow and avoid flow computation during testing.

Unlike traditional knowledge distillation, between the teacher and student networks, we designed a knowledge distillation loss based on semantic graph mapping, which allows the student network to learn heterogeneous knowledge from the teacher network, enabling single-video data features to learn multimodal data features and possess more feature representations, thereby improving the activity recognition performance of the student network.

## Methods

### Overview

Overall, we propose a knowledge distillation architecture with multiple teacher networks and a single student network, as illustrated in Fig. 1. This architecture first learns modal knowledge from wearable sensors through pre-training the teacher networks. Subsequently, the acquired knowledge is transferred to the student network through knowledge distillation, guiding the training of the student network. Finally, the student network achieves a performance similar to that of inputting multimodal data when only video data is provided.

In Fig. 1, we use virtual images generated from one-dimensional time-series data from wearable sensors as inputs to the teacher network. Multiple teacher networks correspond to different types of wearable sensor data. The input to the student network is a sequence of 32 frame images constituting a video frame sequence.

Both the teacher and student networks consist of four stages. After each stage, both modal networks obtain corresponding feature maps. The teacher network transfers heterogeneous knowledge from intermediate layers to the student network through the semantic graph mapping (SGM) module and calculates semantic loss (SE Loss) and soft target loss (ST Loss) at the final stage for training the student network.

**Figure 2:**
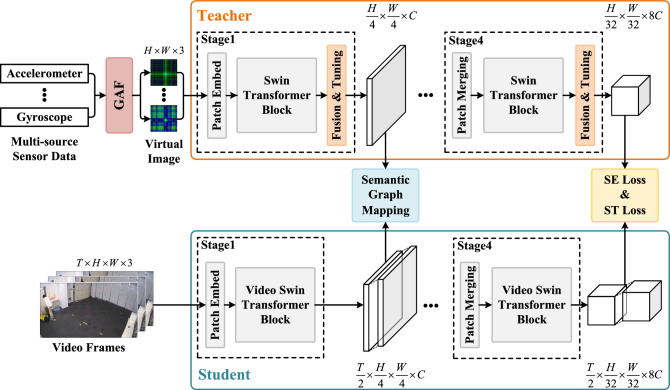
In the teacher network, virtual images are processed using Swin Transformer blocks, while in the student network, video data is processed using Video Swin Transformer blocks. Within the teacher network, features from different types of sensor data are fused and adjusted using the fusion and tuning (FT) module. The teacher network transfers heterogeneous knowledge to the student network through the semantic graph mapping (SGM) module.

### Details of stage

The Fig. 2 provides detailed specifics of each stage. For image recognition, most traditional methods rely on convolutional neural networks (CNNs). However, CNNs have the drawback of a relatively small receptive field during the early stages of feature extraction may lead to the inability of the model to effectively identify detailed features in images at the beginning. Therefore, in the teacher network, we adopt the feature extraction method of the Swin Transformer (Swin), which achieves global understanding through self-attention mechanisms and window-based attention mechanisms that CNNs lack.

For feature extraction in videos, traditional recurrent neural networks (RNNs) or long short-term memory networks (LSTMs) suffer from problems such as forgetting long-term frame relationships, making it difficult to focus on long-term temporal relationships. In the student network, we introduce video swin transformer (Video Swin), which simultaneously considers both temporal and spatial dimensions. This helps the model systematically extract motion information from videos. Both approaches are based on Swin, providing inherent compatibility between them.

Next, we briefly introduce the working principle of Swin Transformer. Given an image $I \in \mathbb {R}^{h\times w\times 3}$, we first divide it into patches of size $p$, and then map each patch to dimensions recognizable by Swin Transformer, denoted as $x \in \mathbb {R}^{H \times W \times C}$, where $H=h/p$, $W=w/p$, and $C=C\times p^2$. Except for the first stage, each stage begins with a patch merging operation to obtain the input for the next Stage Block, denoted as $x^i \in \mathbb {R}^{H/2\times W/2\times 2C}$, where $i$ represents the $i$-th stage, $i>1$, and $H,W,C$ are from the previous stage. After several stages, we obtain our final output.

To leverage the complementarity and consistency among different types of sensor data, we design the Fusion and Tuning (FT) module in the first three stages of the teacher network. This module is used to fuse and adjust features from different types of sensors. First, in the teacher network, we map the virtual images from wearable sensors to feature embeddings of dimensionality through patch embedding. Then, we extract features from different types of data using Swin blocks. Subsequently, through the FT module, we fuse the features from different types of sensors and adjust the original features of different types of data. This process helps us maintain consistency within the data while incorporating complementary knowledge from different types of data.

Since traditional knowledge distillation only transfers knowledge at the final feature output layer, neglecting the rich feature knowledge in intermediate layers, we propose the SGM module to transfer heterogeneous knowledge from the intermediate layer of the teacher network to the student network. In the student network, we first map the video frame sequence to feature embeddings of dimensionality through patch embedding. Then, we perform knowledge distillation between the corrected features from the teacher network and the features of the student network through the SGM module. Subsequently, the feature maps of each network undergo patch merging before entering the next block. Finally, SE loss and ST loss calculations are performed on the output features of the last stage to train the student network.

We seamlessly integrate the FT module and the SGM module with the two Swin architectures and transfer heterogeneous knowledge through knowledge distillation. In the final implementation, we achieve both approximate multimodal effects and maintain high accuracy in the absence of data.

### Virtual image generation

The raw sensor data typically only contains motion information, thus concealing time-related details. Relying solely on raw data for analysis overlooks the correlation of each time step, which is not conducive to the final prediction.To preserve local temporal relationships in one-dimensional time series data from wearable sensors and better convey their content to RGB video data, we employed gramian angular field (GAF) to convert the one-dimensional time series data into two-dimensional, three-channel color images.

Since wearable sensor data contain time series signals from three axes (*x*, *y*, *z*), we represent the signal data from one axis as $X=\{x_1, x_2,..., x_n\}$. Then, we normalize the original signal *X* using the min-max normalization method to scale it within the range of $[-1, 1]$, obtaining the normalized signal $\hat{X}$:

1 $$\begin{aligned} \hat{X}=\frac{(X-\text {max}(X))+(X-\text {min}(X))}{\text {max}(X)-\text {min}(X)} \end{aligned}$$

Next, the normalized signal $\hat{X}$ is represented in polar coordinates using a transformation function *f*. Specifically, this involves using the timestamp as the radial coordinate $\rho$, and expressing the normalized signal $\hat{X}$ as the polar angle $\theta$ in the range $[0, \pi ]$. Ultimately, the one-dimensional normalized signal $\hat{X}$ is transformed into two-dimensional polar coordinates $(\rho , \theta )$:

2 $$\begin{aligned} f(x_i,t_i,N)= {\left\{ \begin{array}{ll} \theta _i= \arccos (\hat{x_i}), &{}x_i\in \hat{X} \\ \rho _i = \frac{t_i}{N} \end{array}\right. } \end{aligned}$$

Where $\hat{x_i}$ represents the normalized temporal signal, $t_i$ denotes the temporal position of the signal, and *N* represents the number of data points in the temporal signal. After encoding the one-dimensional time series signal into polar coordinates, the calculation of the sum of triangles between points facilitates the easy derivation of the correlation coefficient between two points within local time. As correlation coefficients can be computed using the cosine of the angle between vectors, the correlation between time *i* and time *j* can be expressed as $\cos (\theta _i + \theta _j)$ . Consequently, we obtain the GAF correlation matrix *G*:

3 $$\begin{aligned} G=\begin{pmatrix} \cos (\theta _1 + \theta _2) &{} \dots &{} \cos (\theta _1 + \theta _n) \\ \vdots &{} \ddots &{} \vdots \\ \cos (\theta _n + \theta _1) &{} \dots &{} \cos (\theta _n + \theta _n) \end{pmatrix} \end{aligned}$$

Since $\cos (\theta )$ is uniquely determined within the interval $\theta \in [0, \pi ]$, this implies that the method can retain the characteristics of the original data. By computing the correlation matrix, it is possible to augment the local temporal relationships lacking in the original data.

This approach can preserve local temporal relationships in the form of temporal correlations as the number of timestamps increases. The wearable sensor data is formatted as three-axis time series (x, y, and z axes), assuming a time length of *n* for each axis. Using the aforementioned method, we obtain a $n \times n$ GAF matrix for each axis. Subsequently, the GAF matrices of the three axes are concatenated along the channel dimension to form a three-channel image of size $n \times n \times 3$, denoted as $P = \{ G_x, G_y, G_z \}$.

Through this method, we successfully convert one-dimensional time series data into a two-dimensional image format, followed by the stacking of the images of the three axes to obtain the final three-dimensional image format. This RGB image generated by encoding sensor data serves as the input to the teacher network.

**Figure 3:**
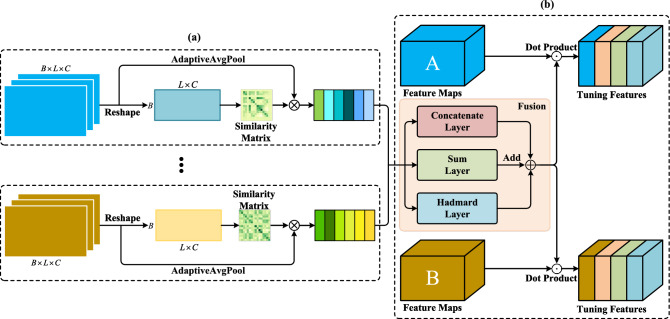
Fusion and tuning (FT) module in the teacher network. A and B represent the output features after the stage layer in the network. (**a**) Intra-modality global context modeling: constructing global models for feature maps to obtain global information of the feature maps. (**b**) Feature Fusion and Adaptive: fusion of different types of global feature information in multiple ways and using it to calibrate the original feature maps for subsequent feature extraction.

### Fusion and tuning

In the structure of the teacher network, we employ the Swin Transformer (Swin) as the feature extractor. Due to its utilization of global attention mechanisms, the Swin Transformer can better capture the global relationships within the data compared to traditional convolutional neural networks. However, for different types of sensor data, using the same backbone will result in different attention feature matrices. Existing attention mechanisms fail to adequately focus on the correlations between features from different types of sensors, often overlooking such correlations during multi-source feature fusion. To fully exploit the correlation information among different types of data, we integrate the similarity within features and the correlation knowledge between multiple sensor features between each Swin-Stage module. Subsequently, the fused activation signals are used to adjust the features of various sensor types. The similarity fusion module between two types of sensors is illustrated in Fig. 3.

#### Intra-source global context modeling

Incorporating the FT module into each stage module of Swin for feature fusion of multi-source data.Suppose there are *m* types (e.g. different types of sensor data), and the backbone network consists of *s* Stage modules. Given an input batch size of *B*, the output feature maps $F_m^s$ of different types of data *m* at a specific Stage block *s* in the network are represented as $F_m^s\in \mathbb {R}^{B\times L\times C}$, where *B* is the batch size, *L* is the number of patches, and *C* is the feature dimension per patch. Inspired by attention-based knowledge transfer methods^51,53^, this approach utilizes activation correlation for knowledge transfer. First, reshape the feature maps $F_m^s$ into $R_m^s\in \mathbb {R}^{B\times L\cdot C}$, then perform matrix multiplication with its transpose and L2 normalization to obtain the similarity matrix within each type of data, denoted as $G_m^s\in \mathbb {R}^{B\times B}$.

4 $$\begin{aligned} \hat{R}_m^s= & {} R_m^s \times R_m^{s\mathsf T} \end{aligned}$$

5 $$\begin{aligned} G_m^s= & {} \frac{\hat{R}_m^s}{ \begin{Vmatrix} \hat{R}_m^s \end{Vmatrix}_2} \end{aligned}$$

where $$\begin{Vmatrix} \cdot \end{Vmatrix}_2$$ represents the L2 norm. After obtaining the similarity matrix within the data, we use it to guide the construction of global context within the data. First, we employ one-dimensional adaptive average pooling to compress the feature maps outputted by the Stage, obtaining the compressed feature vectors $V_m^s\in \mathbb {R}^{B\times C}$. Then, we perform matrix multiplication between the similarity matrix $G_m^s$ and the compressed feature vectors $V_m^s$ to obtain the global context information within the modality $S_m^s \in \mathbb {R}^{B\times C}$, with the calculation formula as follows:

6 $$\begin{aligned} V_m^s(B,C)=\frac{1}{L}\sum _{i=1}^{L}F_m^s(B,i,C) \end{aligned}$$

7 $$\begin{aligned} S_m^s=G_m^s \times V_m^s \end{aligned}$$

#### Feature fusion and tuning

The global context information $S_m^s$ include similarity features within the data, which can effectively reflect the characteristics of each type of data. To fully exploit the correlation and complementary information between different types of data, we employ three joint representation forms for the global context information $S_m^s$ across different types: concatenation, summation, and Hadamard product. These methods have been proven to be effective in previous studies^54^. Below are the formulas for generating the three joint representation activation signals:

8 $$\begin{aligned} E_{con}^s= & {} W_{con}^s[S_1^s, \dots ,S_m^s]+b_{con}^s \end{aligned}$$

9 $$\begin{aligned} E_{sum}^s= & {} W_{add}^s \left( \sum _{i=1}^m S_i^s \right) +b_{sum}^s \end{aligned}$$

10 $$\begin{aligned} E_{had}^s= & {} W_{had}^s \left( \prod _{i=1}^m S_i^s \right) +b_{had}^s \end{aligned}$$

where $[\cdot ,\cdot ]$ represents concatenation operation, $\prod _{i=1}^m$ denotes element-wise multiplication (Hadamard product) operation on the global context information, *W* and *b* are learnable parameters, $[W_{con}^s,b_{con}^s],[W_{sum}^s,b_{sum}^s],[W_{had}^s,b_{had}^s]$ respectively represent the weights and biases of the concatenation, summation, and Hadamard product layers. These three joint representation layers scale the channel features of $S_m^s$ and apply the GELU activation function after the scaling operation to enhance the model’s generalization capability.

After obtaining the activation signals of the three aggregated global context information, we use them to correct the original feature maps to incorporate complementary information from different types. We use the GELU activation function to introduce non-linearity to the activation signals, then simply add them to obtain the fusion knowledge for correcting the original type features. The formulas for correcting different type features are as follows:

11 $$\begin{aligned} \hat{F}^s_m=(GELU(E_{con}^s)+GELU(E_{sum}^s)+GELU(E_{had}^s)) \odot F_m^s \end{aligned}$$

where $\odot$ denotes element-wise multiplication operation in the *C* dimension. Through the FT module, we can achieve multi-source fusion of attention features and re-adjustment of features of different types, allowing different types of features to calibrate each other while maintaining the similarity within the data and the correlation between different types of data.

**Figure 4:**
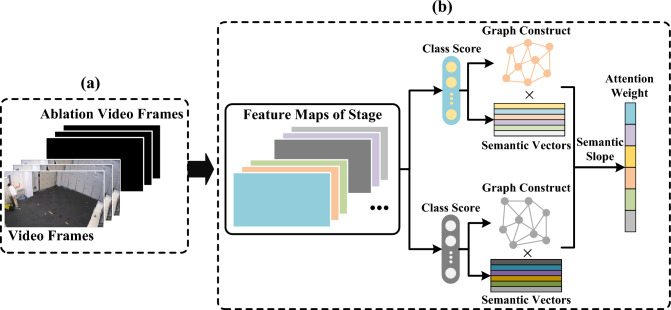
The SGM module in the intermediate layers. (**a**) Concat Data: The combination of original video data with its ablated data is used as the input to the network. (**b**) semantic graph mapping: Based on the predicted semantic embeddings, the attention weight changes of the feature maps at the intermediate stage layers are obtained.

### Semantic graph mapping

Traditional knowledge distillation methods typically transfer knowledge at the final feature output layer, overlooking the rich information in the intermediate layers. When dealing with heterogeneous data such as motion sensors and visual sensors, the complementary information in the intermediate layers is crucial for transferring heterogeneous knowledge. To better achieve heterogeneous knowledge transfer from the teacher network to the student network, we designed a semantics-based knowledge distillation module, which operates on the first s-1 stage modules to generate semantic-aware attention heatmaps for the intermediate layers of the teacher and student networks, aiming to train the student network accordingly. The objective of this module is to highlight important semantic regions in the output feature maps of each Stage and train the student network based on the differences between the teacher and student networks.

Previous research^55^ has demonstrated that ablating certain units in a model can assess the importance of those units for specific category recognition. Therefore, this paper combines original images with their channel-zeroed ablated data to generate semantic ablation decreases, obtaining a good multimodal semantic visual interpretation through semantic ablation decreases.

We take the student network as an example to explain the operation principle of the SGM module in Fig. 4. During the training of the student network, the student network takes a combination of the original data $I \in \mathbb {R} ^{B\times C\times D\times H\times W}$and the ablated data $I_a \in \mathbb {R}^{B\times C\times D\times H\times W}$ with zeroed channel values as input, denoted as combined data $[I;I_a]\in \mathbb {R}^{2B\times C\times D\times H\times W}$. Firstly, we obtain the network’s classification prediction and find the corresponding semantics. Let *y* represent the predicted category of the original data, and $y_a$ represent the predicted category of the ablated data. Then, we utilize Bert^56^ to compute the semantic embeddings for the original data*B* and the ablated data $B_a$:

12 $$\begin{aligned} \begin{aligned} B&= Bert(y) \in \mathbb {R}^{B\times 768}\\ B_a&= Bert(y_a) \in \mathbb {R}^{B\times 768} \end{aligned} \end{aligned}$$

Next, leveraging manifold learning^57^ which can extract intrinsic structures from data, we constructed two graphs for the class score vectors of *I* and $I_a$. In these graphs, vertices represent feature vectors used for classification, while edges represent relationships between feature vectors, determined by Gaussian similarity. Assuming the input feature vectors are $x_i$ and $x_j$, the weight of the edge between them is given by:

13 $$\begin{aligned} W_{i,j}=exp\left( -\frac{ \begin{Vmatrix} x_i-x_j \end{Vmatrix}^2}{2}\right) \end{aligned}$$

Once we obtained the graph structure *W*, we introduced normalized graph Laplacians^58^ and applied them to *W*, which helps further extract the internal partitioning features of *W*, i.e. $L=D^{-\frac{1}{2}}WD^{-\frac{1}{2}}$. Here, *D* is a diagonal matrix where the value on the diagonal of the $i^{th}$ row is equal to the sum of the $i^{th}$ row of *W*. This way, the manifold structure of the data can be effectively represented in the graph matrix $L\in \mathbb {R}^{B\times B}$.

To preserve the structure of the original features, we first multiply the semantic embeddings by the graph matrix, which is essentially a form of manifold regularization. Next, we define a slope metric $k\in \mathbb {R}^{B\times 768}$ to measure the rate of semantic change from the original data to the ablated data, and based on this metric, we allocate attention weights to the feature maps. The computation of *k* is given by Eq. (14):

14 $$\begin{aligned} k = \frac{L\times B-L_a\times B_a}{L\times B} \end{aligned}$$

where *L* and $L_a$ are the normalized graph similarity matrices of the original data and the ablated data, respectively. By computing the rate of semantic change, we can represent the importance values of features in terms of the magnitude of the slope while preserving the essence of the data. Finally, we perform a Hadamard product operation between the semantic change rate and the original features to obtain the semantic graph mapping, which will be used for the loss calculation in knowledge distillation.

15 $$\begin{aligned} M_m^s=LN(k\cdot F_m^s) \end{aligned}$$

where $LN(\cdot )$ represents the LayerNorm function^59^. We utilize the semantic graph mappings generated by the teacher network as soft targets, along with those generated by the student network, to construct the SGM knowledge distillation loss. Assuming we have *m* teacher modalities and one student modality with *s* Stage modules, we employ the mean squared error (MSE) loss of SGM to transfer cross-modal heterogeneous knowledge.

16 $$\begin{aligned} L_{SGM}=\frac{\sum _{i=1}^{s-1}\sum _{j=1}^{m}MSE(M_S^i,M_j^i)}{m\times (s-1)} \end{aligned}$$

where we only utilize the last $s-1$ stage modules to compute the SGM loss, and the last module is used for calculating the semantic loss; $M_S^i$ represents the semantic graph mapping of the *i*-th Stage of the student network.

### Training

Our framework is a multi-scale knowledge distillation architecture. We utilize a sensor image generation model to convert one-dimensional time-series sensor data into RGB image data recognizable by Swin through GAF, thereby preserving the original features while enhancing local temporal relationships for subsequent feature extraction. After the attention computation is completed in the Stage modules of the teacher network, the feature maps are input into the Fusion and Tuning (FT) module to obtain similarity features of different types of wearable sensor data and perform multi-type feature fusion. During the training of the student network, we use the Semantic Graph Mapping (SGM) module to map the semantic key parts of intermediate feature maps and calculate knowledge distillation loss using the semantic feature maps of the teacher network as soft targets. We seamlessly integrate these modules into the Swin knowledge distillation architecture to address the heterogeneous HAR problem between sensor data and visual data.

We first independently train the teacher network, as shown in Fig. 2, using Swin as the backbone network to construct the teacher network, which primarily takes sensor image data as input and undergoes fine-tuning training on Swin with pre-trained weights. After the training of the teacher network is completed, it guides the training of the student network. Our ultimate goal is to use the student network, with video data as input, to generate predictions similar to those trained with multimodal data. The teacher network’s fused features are used together with the features learned by the student network for computing the semantic graph mapping loss, thereby achieving knowledge transfer.

To train the teacher network, we use the output features of the last stage as the semantic feature output of the entire network and compute the mean squared error (MSE) loss between it and the target true semantic embeddings, which we refer to as the semantic loss. Assuming we have m teacher modalities, the total loss of the teacher network, combined with semantic loss and predicted cross-entropy loss, is shown in Eq. (17):

17 $$\begin{aligned} L_{SE}= & {} \frac{1}{m}\sum _{i=1}^m MSE(F_i,B_i) \end{aligned}$$

18 $$\begin{aligned} L_T= & {} \frac{1}{2\times m}\left( \sum _{i=1}^m MSE(F_i,B_i)+\sum _{i=1}^m CE(P_i,T_i)\right) \end{aligned}$$

where $F_i$ represents the semantic features of modality *i* in the network, $B_i$ represents the true semantic embeddings, $P_i$ represents the model’s predicted results, $T_i$ represents the ground truth labels, $MSE(\cdot )$ denotes the mean squared error loss function, and $CE(\cdot )$ denotes the cross-entropy loss function.

After the training of the teacher network is completed, we utilize the knowledge of the teacher network to train the student network. The loss of the student network includes semantic loss, predicted cross-entropy loss, SGM knowledge distillation loss, and soft target knowledge distillation loss. Particularly, the soft target knowledge distillation loss focuses on the differences in similarity of classification score features and utilizes KL divergence for loss computation. The specific formula is as follows:

19 $$\begin{aligned} L_{ST}^S= & {} \frac{1}{m}\sum _{i=1}^m KL\left( \frac{P_i}{T},\frac{P_S}{T}\right) \end{aligned}$$

20 $$\begin{aligned} L_S= & {} \alpha L_{ST}^S+\beta L_{SGM}^S+\gamma L_{SE}^S+L_{CE}^S \end{aligned}$$

where $\alpha , \beta , \gamma$ are hyperparameters, we set $\alpha =0.1, \beta =1, \gamma =1$ and *T* is the temperature parameter in the KL divergence, $L_{ST}^S$ is the soft target loss of the student network, $L_{SGM}^S$ is the semantic graph mapping loss of the student network, $L_{SE}^S$ is the semantic loss of the student network, and $L_{CE}^S$ is the predicted cross-entropy loss of the student network. We train the student network with the total loss of the student network.

## Results and discussion

### Experimental setup

#### Datasets

We evaluated the performance of our model on two multimodal activity datasets: UTD-MHD and MMAct. UTD-MHAD and MMAct are both from public datasets and no living beings, humans, or animals were involved. MMAct is a large multimodal action dataset consisting of 35 different activities performed by 20 volunteers, totaling over 36,000 samples. These data include information from various modalities such as RGB videos, keypoints, depth images, and signals from wearable sensors. Each activity has 5 different executions and 4 different scenarios recorded from four different directions using visual sensors. In this study, we primarily utilized RGB videos and wearable sensor data (including smartphone accelerometers, smartwatch accelerometers, gyroscopes, and orientation information) from the MMAct dataset. The UTD-MHAD dataset comprises 27 activities performed by 8 volunteers. Each volunteer repeated each activity 4 times, and their wearable sensor data and video data were recorded. This dataset contains a total of 861 samples, each including RGB videos, depth images, skeleton information, and inertial sensor data. In our research, we mainly used RGB videos and inertial sensor data from the UTD-MHAD dataset, including accelerometer and gyroscope data.

#### Implementation details

For both the MMAct and UTD-MHAD datasets, we adopted the same model setup. We employed the Swin-Transformer Tiny (Swin-T) with pre-trained weights as the backbone network for the teacher network, while for the student network, we used the Video Swin-Transformer Tiny (Video Swin-T) with pre-trained weights as the backbone network. During the training process of both teacher and student networks, we did not use pre-trained weights for the classification heads but fine-tuned the original backbone network weights. When training the teacher network, we resized the sensor virtual images to $224 \times 224$, set the learning rate to 1e-4, batch size to 32, and utilized the AdamW optimizer^60^. When training the student network, we uniformly sampled 32 frames from the original video data, resized them to $224 \times 224$ resolution, set the learning rate to 5e-5, batch size to 16, and similarly used the AdamW optimizer for training. To obtain semantic representations across different modalities, we utilized a Bert model to obtain 768-dimensional semantic vectors corresponding to each action name. For the MMAct dataset, we followed the data split standard of MMAct for training and used F1-score as the evaluation metric. For the UTD-MHAD dataset, we used Accuracy as the evaluation metric. The training of the teacher network was completed on an NVIDIA RTX 3090 GPU, while the training of the student network was conducted on two NVIDIA A40 GPUs.

**Table 1:** Comparison with other models on MMAct and UTD-MHAD datasets.

(a) MMAct (cross-session)		
Method	Modality	F1-score (cross-session)
TSN^34^	RGB	69.20
TRN^35^	RGB	71.95
MMD^4^	A+G+O+RGB	74.58
MMAD^4^	A+G+O+RGB	78.82
Keyless^61^	A+G+O+RGB	81.11
SAKDN^62^	A+G+O+RGB	82.77
HAMLET^63^	A+G+O+RGB	83.89
MuMu^64^	A+G+O+RGB	87.50
**Ours**	**A+G+O+RGB**	**88.12**

(b) MMAct (cross-subject)		
Method	Modality	F1-score (cross-subject)
TSN	RGB	59.5
MMD	RGB	64.33
MMAD	A+G+O+RGB	64.45
TRN	A+G+O+RGB	66.56
HAMLET	A+G+O+RGB	69.35
Keyless	A+G+O+RGB	71.83
MuMu	A+G+O+RGB	76.28
SAKDN	A+G+O+RGB	77.23
**Ours**	**A+G+O+RGB**	**79.52**

(c) MMAct (cross-scene)		
Method	Modality	F1-score (cross-scene)
TSN	RGB	51.21
TRN	RGB	60.03
MMD	A+G+O+RGB	62.23
MMAD	A+G+O+RGB	64.12
Keyless	A+G+O+RGB	70.77
HAMLET	A+G+O+RGB	71.59
SAKDN	A+G+O+RGB	73.48
MuMu	A+G+O+RGB	74.57
**Ours**	**A+G+O+RGB**	**80.08**

(d) UTD-MHAD		
Method	Modality	Accuracy
TSN	RGB	92.54
Keyless	A+G+O+RGB	92.67
MCRL^65^	A+G+O+RGB	93.02
PoseMap^66^	A+G+O+RGB	94.51
TRN	RGB	94.87
HAMLET	A+G+O+RGB	95.12
MuMu	A+G+O+RGB	97.60
SAKDN	A+G+O+RGB	98.60
**Ours**	**A+G+O+RGB**	**98.89**

Significant values are in bold.

### Comparison

We evaluate the performance of our model by comparing it with state-of-the-art HAR methods on two datasets. On the MMAct dataset, we evaluate according to MMAct’s evaluation settings (cross-session, cross-subject, and cross-scene) using F1-Score. Cross-session denotes partitioning based on repeated actions, as shown in Table 1a, where our model achieves a high recognition accuracy when observing all actions, outperforming the recently proposed MuMu model by 0.62%. MuMu is an attention-based collaborative learning model, which first classifies activities by grouping them and then further divides them, but it lacks fusion of intermediate feature similarities, which is why our model is superior. Cross-subject denotes partitioning based on volunteers, as shown in Table 1b. Unlike cross-session partitioning, this partitioning mainly focuses on recognizing actions performed by different individuals, enhancing the model’s robustness to unfamiliar actions. The results show that our model improves by 2.29% compared to SAKDN. The SAKDN model is a convolution-based knowledge distillation network that may not effectively focus on global information during feature extraction, which could lead to a localized representation of subsequent feature information. In contrast, our model extracts global feature information attentively, allowing for a better analysis of unfamiliar actions from a global perspective, thus enhancing action recognition robustness. Cross-scene denotes partitioning based on different scenes, as shown in Table 1c. Surprisingly, we found a 5.51% improvement in our model’s performance under environmental changes, further demonstrating the robustness of the global attention mechanism to complex environments.

In addition to the MMAct dataset, we also evaluated our model’s performance on the UTD-MHAD dataset as shown in Table 1d. Due to the smaller size and simpler actions of the UTD-MHAD dataset, without complex scenes or occlusions, the activities performed by volunteers are simple but distinctive. Although the recognition accuracy of all models is satisfactory, our proposed model still achieves the highest accuracy, with a performance improvement of 0.29.

We also compared with other attention-based methods such as Keyless and HAMLET. These attention-based methods do not consider how to better handle motion sensor data to represent motion information better. On the other hand, non-attention-based methods cannot effectively focus on global information during feature generation in intermediate layers, resulting in lower performance compared to models using a global attention mechanism. The performance drop in Table 1b and c may be due to environmental changes requiring the model to reconsider, dispersing some attention, thus unable to focus solely on activity recognition.

Based on the experimental results on the MMAct and UTD-MHAD datasets, we draw the following conclusions:

- Fusion of complementary information from different types of sensor data can better unify feature information across different types of data.
- Using a global attention mechanism can better acquire global information, thereby extracting better global features.
- Semantic embeddings can serve as cues between different modalities to increase their correlation.

### Ablation study

To assess the contributions of the FT, SGM, SE, and ST modules, we conducted ablation experiments by excluding each of these modules individually. BaseLine: The student network (Video Swin-T) was trained using only RGB videos. W/O FT: Fusion and Tuning were not used in the teacher network. W/O SGM: Semantic Graph Mapping Loss calculation was not performed when transferring knowledge from the teacher network to the student network. W/O SE: Semantic embeddings were not included during the training of both the teacher and student networks. W/O ST: Soft target loss calculation was not performed during the training of the student network.

From the results in Table 2, it can be observed that compared to the ST module, the SGM module contributes more significantly to the overall model, indicating that the semantic graph mapping loss is very helpful for knowledge transfer between the two networks. This also demonstrates the benefits of using the classification score feature vectors from the teacher network as soft targets for knowledge transfer. The FT module also plays an important role throughout the network. Through the FT module, we can calibrate the features between different types of sensors, unifying the data features of different types of sensors. When transferring knowledge to the student network, the FT module enables the teacher network to better transfer knowledge from multiple different types of sensors to the student network. Additionally, semantic features contribute to the joint representation of two different modalities of data. We use the same semantic embeddings as a bridge to jointly represent data from different modalities. By calculating semantic losses between different modalities and within modalities, semantic coherence between different modalities can be unified, helping the model maintain similarity between different modalities. From the results in Table 2, we can conclude that semantic embeddings are helpful for overall model training. It can be observed that compared to other modules, the traditional soft target loss has relatively lower importance for the overall model. This indicates that relying solely on soft targets for guidance cannot effectively transfer knowledge and requires the integration of knowledge from intermediate modules in the network for more comprehensive knowledge transfer.

In our model, we successfully integrated the FT, SGM, and SE modules into the Swin Transformer knowledge distillation framework. During the training of the teacher network, the FT module combines features from different types of sensors to unify the similarity between features. When transferring knowledge to the student network, the SGM module obtains attention weights of semantics between different modalities, transferring heterogeneous knowledge between different modalities. During the training of both the student and teacher networks, the SE module is used to reduce the differences between different modalities of data. The integration of these modules mutually reinforces each other and contributes to the improvement of the model’s performance.

**Table 2:** Ablation analysis of different modules of our proposed architecture under the cross-subject partitioning criterion on the MMAct dataset.

Method	Accuracy
Baseline (video swin-T)	76.87
W/O FT	77.63
W/O SGM	77.06
W/O ST	78.75
W/O SE	78.23
**Ours**	**79.52**

“W/O” denotes without. Significant values are in bold.

**Figure 5:**
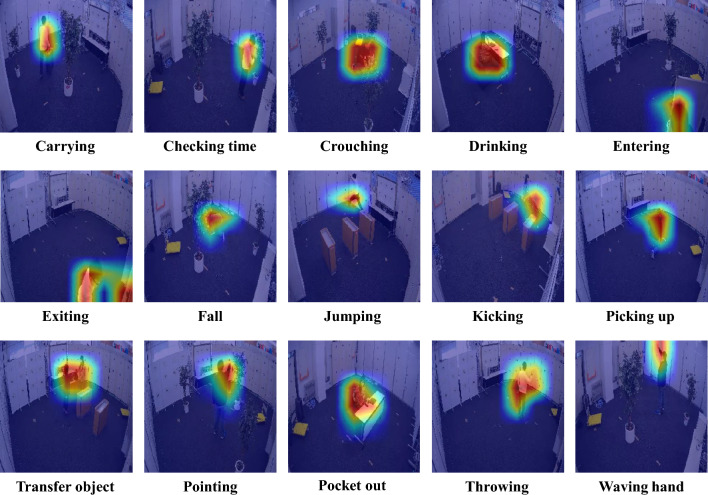
Visualization of the focal regions of interest in MMAct video data by our proposed model.

### Visualization analysis

In our study, to analyze whether the model correctly focuses attention on key locations, we conducted visual analysis of attention heatmaps. We extracted the feature maps from the last stage block output of the student network and its gradients during backpropagation, and used the Grad-CAM method^67^ to calculate the regions in the feature maps that had the most impact on the model’s decision, generating corresponding heatmaps. Specifically, we computed the gradient changes based on the attention feature matrix of video data and averaged them over the time dimension to reflect the key frames in the video data. In Fig. 5, we present the attention heatmaps for 15 actions in the MMAct dataset. It can be observed that our model focuses attention on the activities rather than excessively on the human body or the surrounding environment, demonstrating the effectiveness and robustness of our model.

**Figure 6:**
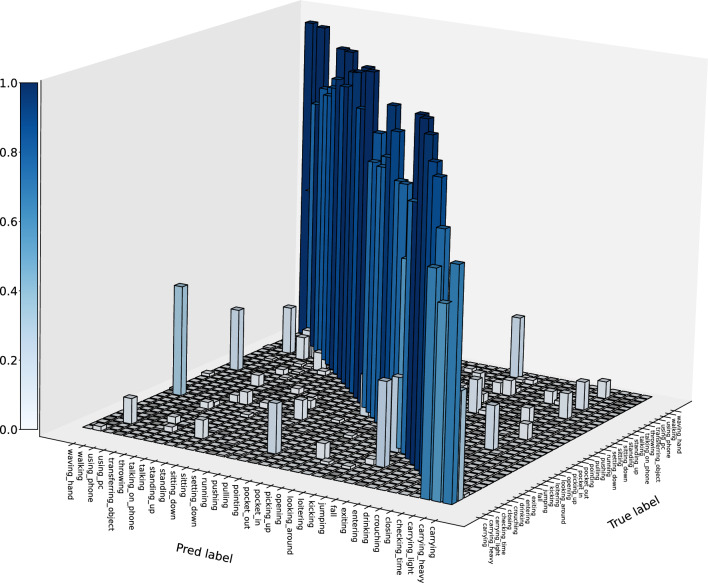
Prediction matrix of our proposed model under the cross-subject partition criterion on the MMAct dataset.

The model prediction analysis based on the cross-subject partition results is illustrated in Fig. 6. The vertical axis represents the true labels, while the horizontal axis represents the predicted labels. We observed that our model performs well in identifying most activities. However, for certain activity categories, the recognition performance is poor, particularly for “carrying,” “jumping,” and “walking.” Specifically, the model struggles to differentiate between “carrying heavy” and “carrying light,” with most samples of “carrying light” being misclassified as the “carrying” category. Upon analysis, these activities exhibit high similarity in visual and motion sensor data features, posing challenges for the model in distinguishing them. Additionally, 22% of “jumping” activities are misclassified as “fall,” and 33% of “walking” activities are misclassified as “loitering” due to similarities in data features. However, for similar activities such as “setting down” and “picking up,” our model demonstrates effective identification, indicating its proficiency in recognizing most similar activities.

Through analysis of the model’s attention heatmap visualization and prediction results, we found that although there are shortcomings in some challenging activity recognition aspects, overall, our model performs better than other models.

## Conclusion

In this paper, we propose a multimodal knowledge distillation architecture. First, we use GAF to convert wearable sensor data into images and use these images as training data input for a multi-type teacher network based on Swin-Transformer. In this network, we perform feature fusion and calibration between types through the FT module. Subsequently, a network of trained teachers is used to mentor the student network. During the training process of the student network, we use the SGM module to transfer the multi-modal knowledge learned by the teacher network to the student network. Experimental results show that our model effectively integrates complementary information between wearable sensors and visual sensors and exhibits good robustness in deployment in complex environments. However, the complexity of global attention calculation is still high, and we will study how to reduce the complexity in the future.

## Data Availability

The original data sets UTD-MHAD^5^ and MMAct^4^ can be obtained according to the address given by the reference. Other data in the article are available upon request from the corresponding author.
